# Metabolic adjustments of blood-stage *Plasmodium falciparum* in response to sublethal pyrazoleamide exposure

**DOI:** 10.1038/s41598-022-04985-7

**Published:** 2022-01-21

**Authors:** Shivendra G. Tewari, Bobby Kwan, Rubayet Elahi, Krithika Rajaram, Jaques Reifman, Sean T. Prigge, Akhil B. Vaidya, Anders Wallqvist

**Affiliations:** 1 Department of Defense Biotechnology High Performance Computing Software Applications Institute, Telemedicine and Advanced Technology Research Center, U.S. Army Medical Research and Development Command, Fort Detrick, MD USA; 2 The Henry M. Jackson Foundation for the Advancement of Military Medicine, Inc., Bethesda, MD USA; 3 Department of Molecular Microbiology and Immunology, Johns Hopkins University, Baltimore, MD USA; 4 Department of Microbiology and Immunology, Center for Molecular Parasitology, Drexel University College of Medicine, Philadelphia, PA USA

**Keywords:** Parasite physiology, Malaria, Biochemical networks

## Abstract

Due to the recurring loss of antimalarial drugs to resistance, there is a need for novel targets, drugs, and combination therapies to ensure the availability of current and future countermeasures. Pyrazoleamides belong to a novel class of antimalarial drugs that disrupt sodium ion homeostasis, although the exact consequences of this disruption in *Plasmodium falciparum* remain under investigation. In vitro experiments demonstrated that parasites carrying mutations in the metabolic enzyme PfATP4 develop resistance to pyrazoleamide compounds. However, the underlying mechanisms that allow mutant parasites to evade pyrazoleamide treatment are unclear. Here, we first performed experiments to identify the sublethal dose of a pyrazoleamide compound (PA21A092) that caused a significant reduction in growth over one intraerythrocytic developmental cycle (IDC). At this drug concentration, we collected transcriptomic and metabolomic data at multiple time points during the IDC to quantify gene- and metabolite-level alterations in the treated parasites. To probe the effects of pyrazoleamide treatment on parasite metabolism, we coupled the time-resolved omics data with a metabolic network model of *P. falciparum*. We found that the drug-treated parasites adjusted carbohydrate metabolism to enhance synthesis of myoinositol—a precursor for phosphatidylinositol biosynthesis. This metabolic adaptation caused a decrease in metabolite flux through the pentose phosphate pathway, causing a decreased rate of RNA synthesis and an increase in oxidative stress. Our model analyses suggest that downstream consequences of enhanced myoinositol synthesis may underlie adjustments that could lead to resistance emergence in *P. falciparum* exposed to a sublethal dose of a pyrazoleamide drug.

## Introduction

The malaria parasite *Plasmodium falciparum* is the causative agent of the most severe form of malaria and is responsible for 97% of malaria-related deaths globally^1^. Due to worldwide efforts, malaria-related deaths have decreased from 736,000 in 2000 to 409,000 in 2019^2^, however, this decrease in malaria mortality rate has plateaued in the last five years^2^. Treatment to decrease malaria-related deaths depends on the use of drugs that have been losing efficacy due to drug resistance^3^. In fact, *P. falciparum* has developed some degree of resistance to all approved antimalarial drugs to date^3^, forcing development of drug-combination therapies involving two (or more) drugs to treat malaria and, hopefully, also suppress drug-resistance development. As emergence of resistance to both single and combination therapies continues^4^, there is an unrelenting need to identify novel antimalarial drug targets.

One such global effort led to the identification of a P-type ATPase, PfATP4, which appears to be the target of multiple drugs of different chemical classes. The PfATP4-associated compounds, such as pyrazoleamides and spiroindolones, disrupt sodium ion homeostasis and cause multiple morphological and physiological changes^5,6^. *P. falciparum* carrying point mutations in PfATP4 have differential sensitivity to spiroindolone and pyrazoleamide compounds, e.g., the A211T mutation in PfATP4 increases the 50% inhibitory concentration (IC_50_) to GNF-Pf4492, a pyrazoleamide compound, but decreases the IC_50_ to KAF246, a spiroindolone compound^7^. How PfATP4 mutations are associated with differential sensitivity of drugs against *P. falciparum* is unclear. Prolonged exposure to sublethal concentrations of PfATP4-targeting drugs induce drug-resistant mutations in the gene and loss of efficacy against *P. falciparum*^8–10^. Similarly, pyrazoleamides induce drug-resistant mutations in the PfATP4 gene^7,10^. Here, we aim to understand the immediate metabolic adjustments of previously unexposed *P. falciparum* in response to a 48-h sublethal exposure to PA21A092. This adjustment constitutes the immediate survival phenotype and highlights the ability of the parasite to survive stress induced by the disruption of sodium ion homeostasis.

Towards this end, we first identified a dose of PA21A092 that caused a significant reduction in growth of *P. falciparum* Dd2 strain over a single intraerythrocytic developmental cycle (IDC). At this drug concentration, we did not observe any changes in morphologies of the treated parasites, suggesting that the identified sublethal dose did not cause major physiological alterations in *P. falciparum*. To characterize pyrazoleamide-induced perturbations in *P. falciparum* metabolism, we collected transcriptomic and metabolomic data at multiple time points during the IDC and coupled these data with a genome-scale metabolic model of *P. falciparum*^11,12^. Our model-based analyses revealed metabolic adaptations in *P. falciparum* that can counter the effects of sodium ion homeostasis disruptors, such as PA21A092. The identification of *Plasmodium* enzymes and pathways associated with drug-resistance development would allow development of rationally motivated drug-combination therapies that can mitigate evolution of drug-resistant progenies of *P. falciparum*.

## Results

### Pyrazoleamide-treated P. falciparum cultures

**Figure 1:**
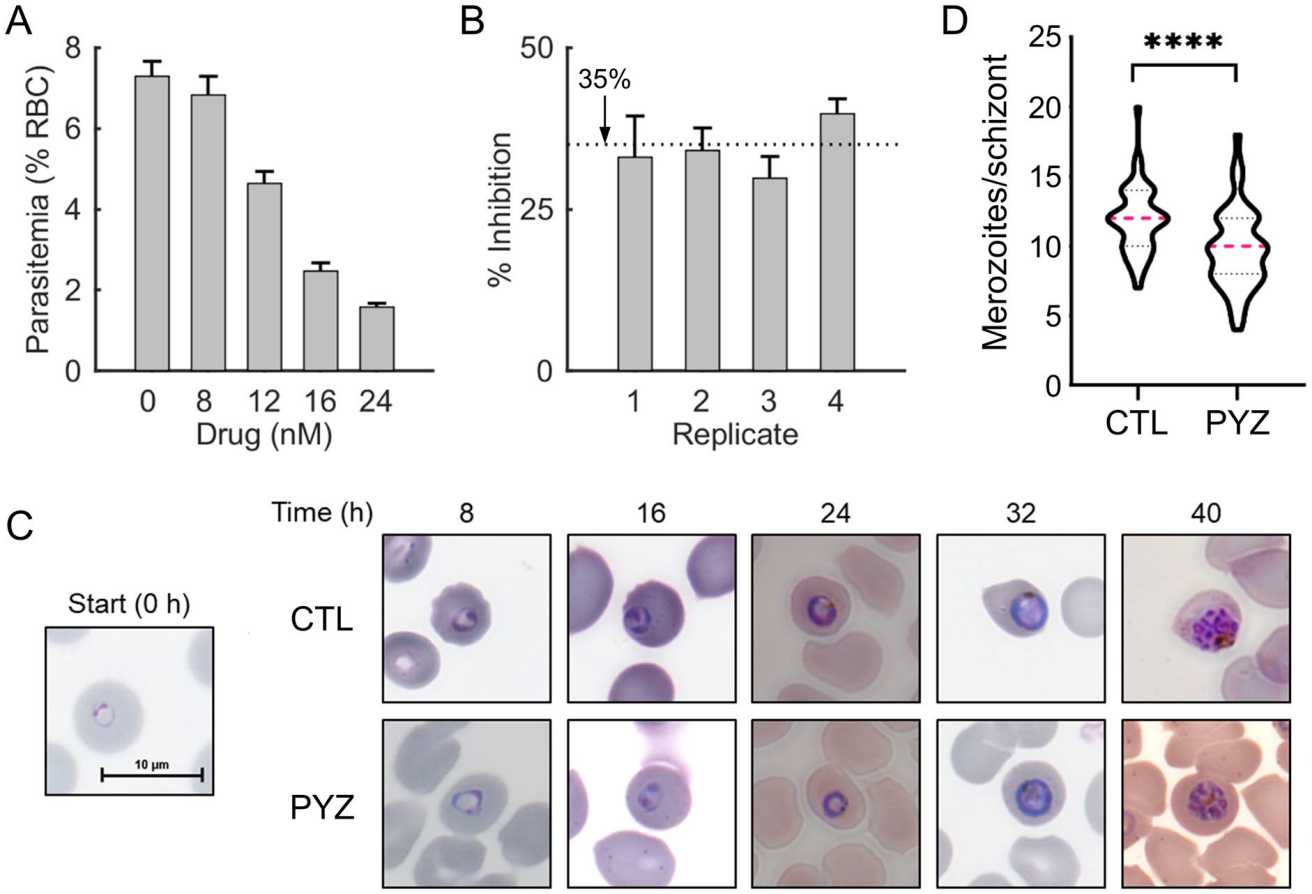
Experiments to determine a sublethal dose of PA21A092, a pyrazoleamide drug. (**A**) Parasitemia at ~ 70 h of experiment after treatment with the notated doses of PA21A092. We treated highly synchronized parasite cultures with 0, 8, 12, 16, or 24 nM PA21A092 in quadruplicate-independent experiments for 40 h. We then assessed the ability of these parasites to replicate by cutting the cultures by a factor of 10 with fresh erythrocytes and drug-free medium. The error bars denote one standard deviation from mean parasitemia. (**B**) Inhibition observed in the four pyrazoleamide-treated cultures used for transcriptomic and metabolomic sample collection. The ordinate shows the percentage of inhibition observed in each replicate after treatment with 12 nM PA21A092. For each replicate, the error bars denote variability in triplicate flow-cytometry measurements. The horizontal dotted line denotes the average of percentage inhibition observed across all measurements. (**C**) Representative Giemsa-stained images of untreated (CTL) and drug-treated (PYZ) parasites at the 0-, 8-, 16-, 24-, 32-, and 40-h time points of the experiment. Note that the 0-h time point represents the appearance of > 95% rings in the parasite culture and corresponds to ~ 0–2 h of the experiment. At this time (0 h), we added fresh medium containing the drug (PYZ) or no drug (CTL). (**D**) Number of merozoites per schizont in the untreated and drug-treated parasite cultures at the 40-h time point of the experiment. We found that a 40-h treatment by 12 nM PA21A092 (PYZ) resulted in a significant decrease in daughter merozoites per schizont. Magenta dotted lines represent the median of 50 observations under untreated (CTL) and drug-treated conditions. The black dotted lines denote the 25% and 75% quartiles. The asterisks denote that the difference between median number of merozoites/schizont under the two conditions is statistically significant (*p* < 0.0001; Wilcoxon rank-sum test).

Prolonged culturing of *P. falciparum* in the presence of a sublethal pyrazoleamide dose selects for resistance-conferring mutations in its metabolic enzyme PfATP4^7,10^. To understand the initial metabolic responses of *P. falciparum* in the presence of a pyrazoleamide, we first performed independent experiments in quadruplicate using various doses of PA21A092 to determine the optimal concentration of the drug that will affect parasite growth without killing the parasites prematurely (between 30 and 70% inhibition would be optimal). We treated highly synchronized cultures of ring-stage parasites with different concentrations of PA21A092 for 40 h. We then assessed the ability of these parasites to replicate by cutting the cultures by a factor of 10 with fresh erythrocytes and drug-free medium. Figure 1A shows that 12 nM PA21A092 achieves the desired level of inhibition, therefore, we used this concentration for data-collection experiments.

Prior to sample collection for transcriptomic and metabolomic data profiling, we again quantified the amount of parasite growth inhibition and also monitored the morphologies of untreated and drug-treated parasites during the IDC. Figure 1B quantifies the inhibition of parasite proliferation, as a percentage of parasitemia observed in untreated parasite cultures, confirming that the identified PA21A092 dosage resulted in an inhibition within the desired range (30–70%).

To monitor morphological changes in parasites due to the drug treatment, we performed Giemsa staining every 8 h during the IDC. Figure 1C shows representative images of parasite-infected erythrocytes in the absence and presence of 12 nM PA21A092 at 0, 8, 16, 24, 32, and 40 h of the experiment. These images suggest that the identified dose of PA21A092 did not perturb parasite morphologies substantially until the 40-h time point of the experiment, however, it still caused ~ 35% of the drug-treated parasites to fail to proliferate in the drug-free medium after 40 h (Fig. 1B). We also quantified the number of merozoites per schizont at the 40-h time point under untreated (CTL) and drug-treated (PYZ) conditions. Figure 1D shows that 12 nM PA21A092 caused a significant decrease in the number of daughter merozoites per schizont (CTL: 12; PYZ: 10), suggesting that the drug negatively affected parasite development.

### Transcriptomic changes due to a sublethal dose of a pyrazoleamide

**Figure 2:**
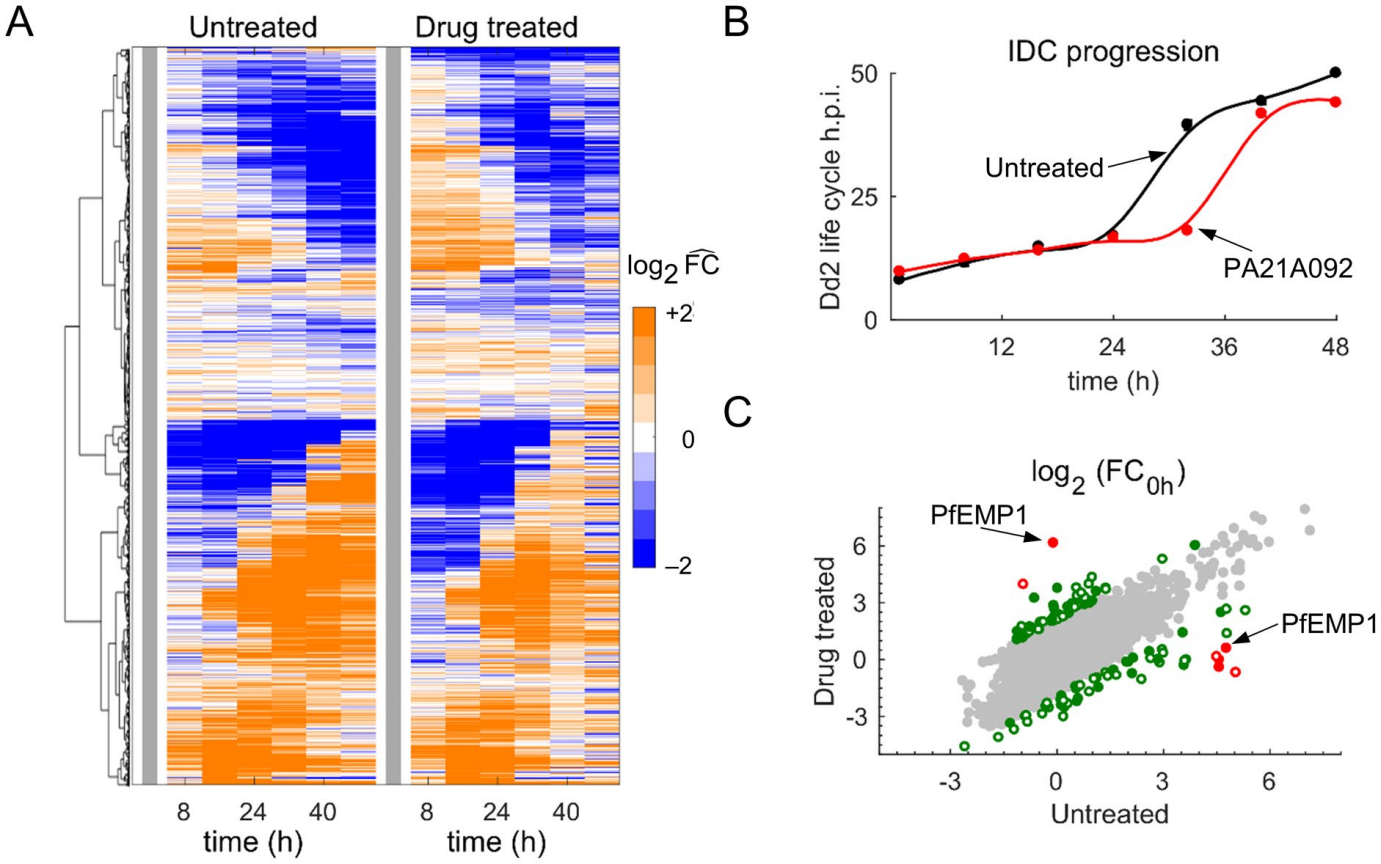
Global analysis of transcriptomic data from untreated and pyrazoleamide-treated *P. falciparum*. (**A**) Hierarchical clustering analysis of transcriptomic data from the untreated and treated parasites. Each row corresponds to a parasite gene, and each column represents a sampled time point of the intraerythrocytic development cycle (IDC). The gene-expression level, under a given perturbation (untreated or drug treated), at a particular time point represents the average of four technical replicates. To facilitate inter-study comparisons, we normalized temporal expression of each gene by its 0-h expression value (vertical gray bars). The untreated and drug-treated parasites appear to have similar expressions during the IDC, indicating a lack of alteration in the parasite’s gene-expression program due to drug treatment. (**B**) IDC progression of untreated and drug-treated parasites. To quantify IDC progression, we computed the Pearson correlation between gene-expression profiles of each replicate from this study and those from the hourly sampled data of Llinas et al.^13^. The closed circles denote the mean of maximum correlation between the two datasets (untreated or treated) at the time points indicated on the *x*-axis. (**C**) Average fold change in gene expression relative to its expression at 0 h (FC_0h_) in untreated and drug-treated parasites. *Plasmodium* genes shown with red circles have fourfold (log_2_ scale) different FC_0h_ after treatment as compared to untreated conditions, while genes shown with gray circles have a less than twofold (log_2_ scale) difference; all other genes are shown using green circles. The open and closed circles denote genes with unknown and known (or putative) functions, respectively. *h.p.i.* hour post infection, *PA21A092* a pyrazoleamide drug, *PfEMP1* *Plasmodium falciparum* erythrocyte membrane protein 1.

To identify major coherent alterations in gene-expression data from the treated parasites, we performed hierarchical clustering analysis (HCA). Figure 2A illustrates the average expression of 5,851 parasite genes during the IDC in the absence and presence of PA21A092. We did not find any major alteration in the transcription program of the drug-treated parasites. To quantify the effect of PA21A092 treatment on *P. falciparum* IDC progression, we compared gene-expression profiles from untreated and drug-treated parasites with those from hourly sampled gene-expression data spanning the entire IDC of *P. falciparum* Dd2 strain^13^.

Figure 2B demonstrates that IDC progression of the drug-treated parasites (red curve) is mostly comparable with that of the untreated parasites (black curve) for the first 24 h, but differing after 32 h into the IDC. Because the expression profile of specific genes may have been affected due to the treatment, we next quantified the average change in expression, relative to 0 h (FC_0h_), of each gene under untreated and drug-treated conditions. Figure 2C shows substantially altered genes in response to drug treatment using red circles and green circles. We found that only eight genes (out of 5,851) had FC_0h_ values that were fourfold (log_2_ scale) different in magnitude after treatment as compared to untreated conditions (Fig. 2C, red circles). Of these eight genes, five encode paralogs of 18S ribosomal RNA, two encode the erythrocyte membrane protein (PfEMP1), and one encodes a conserved *Plasmodium* protein of unknown function (PF3D7_1142900). The PfEMP1 proteins are encoded by a family of approximately 60 *var* genes^14^. An individual parasite only expresses a particular *var* gene at a time, while keeping all other genes transcriptionally silent^14^. Therefore, the dramatically higher expression of one gene and lower expression (PfEMP1, Fig. 2C) of the other is likely due to *var* gene switching and not a result of pyrazoleamide treatment.

To further identify treatment-specific perturbations in the expression of *Plasmodium* genes, we next examined the functional annotation of the genes with a ≥ twofold difference in FC_0h_ values (depicted by green circles in Fig. 2C), and identified 66 (out of a total of 102) genes with known or putative functions (Table S1: Sheet 1). Among these were carbonic anhydrase (PF3D7_1140000) and aquaglyceroporin (PF3D7_1132800). Carbonic anhydrase converts water and carbon dioxide into carbonic acid and protons^15^, while aquaglyceroporin is a water and ammonia transporter of the parasite^16^. The activities of carbonic anhydrase^17^ and aquaglyceroporin^16^ are pH dependent. PfATP4-associated drugs at lethal concentrations are known to alter pH homeostasis in malaria parasites, and these results suggest that a sublethal dose of a pyrazoleamide drug may also cause alterations in the pH of the treated parasites.

To characterize the effects of PA21A092 treatment on *Plasmodium* physiology, we performed an enrichment analysis using gene sets available in the Malaria Parasite Metabolic Pathway (MPMP) database^18^. We included previously published transcriptomic data obtained from *P. falciparum* maintained under three different perturbations^11,12,19^ to isolate treatment-specific effects. Table 1 lists gene sets altered only in pyrazoleamide-treated parasites. Our analysis revealed a substantial downregulation of genes encoding protein kinases (*Cell cycle regulation* in Table 1) and phosphatases (*Phosphatase-related proteins* in Table 1). Both protein kinases and phosphatases play critical roles in phosphorylation and dephosphorylation of regulatory proteins during the IDC^20^.

**Table 1:** Gene sets altered only in pyrazoleamide-treated parasites (We mapped *Plasmodium* genes, with |FC_gene_|≥ twofold, onto 509 gene sets in the Malaria Parasite Metabolic Pathway database^18^).

Gene set description	N_genes_	% of altered genes			
		+ FOS	−APIC	−HXAN	+ PYZ
Cell cycle regulation	53	1.89	1.89	1.89	11.3
Phosphatase-related proteins	38	2.63	2.63	2.63	15.8
LysoPC-sensitive proteins	37	2.70	0.00	0.00	13.5
Mitochondrion electron flow	37	0.00	0.00	2.70	10.8
Tubulin and microtubules	30	0.00	3.33	0.00	13.3
Fatty acid elongation	23	4.35	4.35	4.35	17.4
Centriole proteins	21	4.76	0.00	0.00	14.3
Mitochondrial copper transport	21	0.00	0.00	4.76	14.3

*APIC* apicoplast-disrupted parasites^19^, * + FOS*, fosmidomycin-treated parasites^11^, *-HXAN* hypoxanthine-limited parasites^12^, *LysoPC* lysophosphatidylcholine,*+ PYZ* pyrazoleamide-treated parasites.

FC_gene_ denotes fold change in average expression of a gene due to the perturbation relative to its average expression under normal conditions (see “Materials and methods”).

Although not directly related to our findings, an absence of dephosphorylation processes in *P. falciparum* results in blockage of parasite egress during IDC^21^, suggesting that a sublethal dose of PA21A092 might also be affecting parasite egress during the IDC. Interestingly, a previous study showed premature schizogony-like events in parasites exposed to lethal doses of PfATP4 inhibitors^6^. With a dose of 12 nM PA21A092, we did not observe any premature release of merozoites at any of the time points in our study. Table S2 presents the percentage of altered genes in all the gene sets obtained from the MPMP database^18^.

### Metabolomic data from untreated and pyrazoleamide-treated cultures

**Figure 3:**
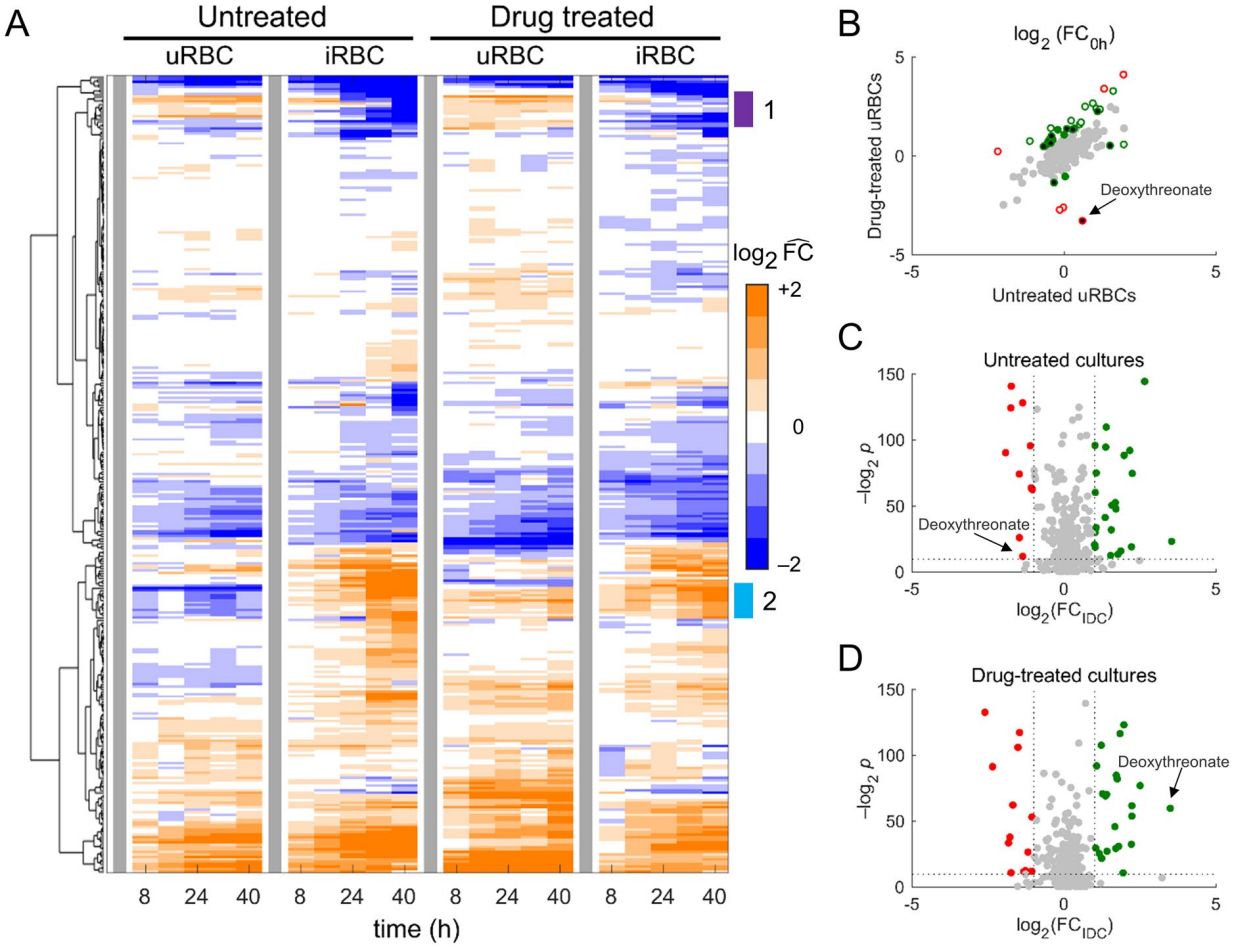
Global analysis of metabolomic data from untreated and pyrazoleamide-treated cultures of uninfected and parasite-infected erythrocytes. (**A**) Hierarchical clustering analysis of metabolomic data from untreated and drug-treated cultures of uninfected (uRBC) and parasite-infected (iRBC) erythrocytes. Each row denotes a metabolite and each column represents a sampled time point during the experiment. The abundance of a metabolite at each time point, under a particular condition (uRBC or iRBC) and perturbation (untreated or drug treated), represents the average of four technical replicates. To facilitate comparisons between different conditions and perturbations, we normalized the abundance of each metabolite by its abundance at 0 h. The vertical gray bars denote the 0-h time point under each condition and perturbation. Clades annotated “1” and “2” suggest alteration in abundance of uRBC metabolites due to the drug treatment. (**B**) Fold change, relative to 0 h (FC_0h_), in abundance of uRBC metabolites under untreated and drug-treated conditions. The uRBC metabolites with |Δ|≥ 4 are shown using red circles, where Δ = ${\text{F}}{\text{C}}_{\text{0h}}^{\text{PYZ}}/{\text{F}}{\text{C}}_{\text{0h}}^{\text{CTL}}$. ${\text{F}}{\text{C}}_{\text{0h}}^{\text{PYZ}}$ and ${\text{F}}{\text{C}}_{\text{0h}}^{\text{CTL}}$ denote FC_0h_ of an uRBC metabolite in the presence and absence of the drug, respectively. The gray circles denote metabolites with |Δ|< 2, while green circles represent metabolites with |Δ|≥ 2 but less than 4. The open and closed circles, respectively, denote metabolites that vary substantially and minimally over a 2-day period^22^. (**C**) Volcano plot showing significantly altered metabolites (*p* < 0.001) due to parasite infection in untreated cultures. The red markers denote significantly altered metabolites with FC_IDC_ ≤ 0.5, while green markers represent significantly altered metabolites with FC_IDC_ ≥ 2; here, FC_IDC_ denotes fold change in average abundance of a metabolite in iRBC cultures relative to uRBC cultures. (**D**) Volcano plot showing significantly altered metabolites (*p* < 0.001) due to parasite infection in drug-treated cultures. The red and green markers, respectively, denote significantly altered metabolites with FC_IDC_ ≤ 0.5 and FC_IDC_ ≥ 2.

We performed HCA on the metabolomic data from untreated and drug-treated cultures of uninfected erythrocytes (uRBC) and parasite-infected erythrocytes (iRBC) to identify drug-induced alterations in *P. falciparum* metabolism. Figure 3A illustrates the average abundance of metabolites detected in quadruplicate samples of uRBC and iRBC cultures. Surprisingly, HCA revealed uRBC metabolites with substantially different abundances under untreated and drug-treated conditions (annotated 1 and 2, Fig. 3A).

To quantify the effect of pyrazoleamide treatment on uRBC metabolite abundances, we computed average fold change in abundance of each metabolite relative to 0 h (FC_0h_) for the untreated and drug-treated datasets. Figure 3B shows metabolites with substantially different FC_0h_ between untreated and drug-treated conditions using red and green circles. In a recent effort, we quantified the expected variance in abundance of uRBC metabolites detected concurrently in distinct donors^22^. Using these expected variances in uRBC metabolite abundances^22^, we found that most of the substantially different metabolites, denoted by open red circles in Fig. 3B, tended to vary in abundance irrespective of a perturbation. Deoxythreonate, shown using closed red circles in Fig. 3B, was primarily detected in urine samples of humans^23,24^ and was not detected robustly (raw count > 1,000; see “Materials and methods”) in our previously analyzed metabolomic datasets^22^. Furthermore, Chen et al.^25^ demonstrated that serum deoxythreonate levels correlate with age of blood donors. We are not aware of the ages of the donors for the independent experiments performed under untreated and drug-treated conditions, but historically the age of the donors for our blood-collection protocol has been between 18 and 60 years.

Because uRBC metabolite abundances can vary between different donors, we normalized the metabolomic data from untreated and drug-treated cultures by an internal standard (see “Materials and methods”) prior to computing fold change in average abundance of metabolites due to parasite infection (FC_IDC_). Figure 3C,D, respectively, show metabolites in untreated and drug-treated cultures that are perturbed significantly (*p* < 0.001) due to parasite infection. Deoxythreonate appears to be depressed (Fig. 3C) or elevated (Fig. 3D) in untreated and drug-treated conditions, respectively, but this is largely due to the variation in its abundance (Fig. 3B) in the uRBC cultures of the two independent experiments.

Table 2 lists FC_IDC_ values of significantly altered metabolites due to the pyrazoleamide treatment. The presence of substantial abundance variations of a specific metabolite in uninfected cultures can either hide or enhance any apparent effect on this metabolite due to the drug treatment^22^. Therefore, in addition to performing two-way analysis of variance (ANOVA), we also computed z-scores of variation in abundances of uRBC metabolites, in the absence and presence of the drug, using their expected variations reported in Tewari et al.^22^. This allowed us to identify uRBC metabolites having significantly different (*p* < 0.001) variation in abundance as compared to their expected variation^22^. In Table 2, we annotated such uRBC metabolites using asterisks. For example, creatinine and hippurate have FC_IDC_ values greater than three in the presence of the pyrazoleamide drug, however, the temporal abundance of both these metabolites in the drug-treated uRBC cultures was significantly different than their expected variation in uRBC cultures^22^. Interestingly, hippurate is a blood metabolic biomarker of gut microbiome diversity in humans^26^.

**Table 2:** List of significantly altered metabolites due to treatment by a pyrazoleamide drug.

Main pathway	Subordinate pathway	Metabolite	FC_IDC_ (SD)^†^	
			CTL	PYZ
Amino acid	Creatine metabolism	Creatinine	0.59 (0.05)	5.73 (0.32)*
	Guanidino and acetamido metabolism	4-Guanidinobutanoate	1.27 (0.07)	0.40 (0.03)
	Histidine metabolism	Imidazole lactate	0.71 (0.04)	3.28 (0.47)
	Phenylalanine metabolism	Hippurate	1.45 (0.11)^§^	3.50 (0.30)*^,§^
Carbohydrate	Glycolysis	Lactate	1.23 (0.14)	2.12 (0.20)
	Pentose phosphate pathway	Sedoheptulose-7-phosphate	0.70 (0.15)	0.20 (0.03)
Energy	Tricarboxylic acid cycle	α-Ketoglutarate	1.90 (0.21)	0.48 (0.05)
		Citrate	1.51 (0.20)	2.25 (0.24)
		Fumarate	1.69 (0.18)^§^	3.40 (0.35)^§^
		Malate	1.47 (0.15)^§^	3.33 (0.26)*^,§^
Lipid	Fatty acid metabolism	4-Deoxythreonate	0.39 (0.05)	11.40 (1.06)
		Hexanoylcarnitine	1.16 (0.09)	0.44 (0.05)
		Pentadecanoylcarnitine^‡^	0.89 (0.04)	0.42 (0.02)
	Phosphatidylserine	1-Palmitoyl-2-oleoyl-GPS (16:0/18:1)	1.42 (0.08)	2.08 (0.27)
	Sphingolipid synthesis	Sphinganine-1-phosphate	1.18 (0.07)	2.39 (0.18)
Nucleotide	Pyrimidine metabolism	Uridine	1.00 (0.14)	2.60 (0.45)
Peptide	γ-Glutamyl amino acid	γ-Glutamylisoleucine^‡^	1.00 (0.08)	0.30 (0.03)
		γ-Glutamylleucine	1.17 (0.08)	0.28 (0.02)

*CTL* uRBC and iRBC cultures maintained in pure RPMI medium, *IDC* intraerythrocytic developmental cycle, *iRBC* parasite-infected RBC, *GPS* glycerophosphoserine, *PYZ* uRBC and iRBC cultures maintained in RPMI medium with a sublethal dose of a pyrazoleamide drug, *RBC* red blood cell, *RPMI* Roswell Park Memorial Institute, *SD* standard deviation, *uRBC* uninfected RBC.

^†^Fold-change (FC_IDC_) values based on a comparison of the average normalized abundance of a metabolite during the IDC in the iRBC culture relative to that in the uRBC culture. The standard deviation (SD) represents deviation from the mean of 10,000 bootstrap samples (see “Materials and methods”) ^**‡**^Metabolite identified based on m/z ratio alone with no external standard for validation. *The temporal variation in metabolite abundance is statistically different (*p* < 0.001) than the variation reported in uRBC cultures in Ref.^22^. ^§^Tewari et al.^22^ found that this metabolite’s abundance changed minimally in uRBC cultures over a 2-day period.

The FC_IDC_ of sedoheptulose-7-phosphate, a pentose phosphate pathway (PPP) metabolite, was smaller in the drug-treated cultures than in the untreated cultures, suggesting a lower production of reduced nicotinamide adenine dinucleotide phosphate (NADPH) due to the treatment. The FC_IDC_ of lactate, a product of the ATP-generating phase of glycolysis, was higher in the drug-treated cultures as compared to the untreated cultures. To further investigate the effect of drug on glycolysis, we compared the temporal profile of glucose and five glycolysis intermediates at the sampled time points between the two conditions. We found that the abundance of these metabolites showed similar alterations up to the 24-h time point, but the values differed considerably afterwards (Table S3). For the untreated cultures, glycolytic intermediates are only 3% of their initial abundances at the 40-h time point, while for the drug-treated cultures, they are 32% of their initial abundances, indicating decreased consumption of these intermediate metabolites (Table S3). Similarly, glycolytic products (pyruvate and lactate) reached 748% of their initial abundance in the untreated cultures, but only 274% of their initial abundance in the drug-treated parasite cultures. Taken together, these alterations suggest a modulation of carbohydrate metabolism in pyrazoleamide-treated parasites, which is consistent with partial inhibition of parasite proliferation during the first IDC.

Although the tricarboxylic acid (TCA) cycle is not essential during the IDC^27^, we found significant alterations in abundance of several TCA cycle metabolites (α-ketoglutarate, citrate, fumarate, and malate). We also found that FC_IDC_ of sphinganine-1-phosphate (Sa1P), an analogue of sphingosine-1-phosphate (S1P), in the treated cultures was almost twice as large as that in the untreated cultures. An increased abundance of S1P is associated with an increase in glycolysis in iRBCs^28^ and, hence, rationalizes the approximately twofold increase in FC_IDC_ of lactate in the treated cultures as compared to the untreated cultures. However, these results do not explain how an increased lactate abundance is associated with a reduced PPP flux in the treated parasites.

Therefore, to identify mechanistic consequences of pyrazoleamide treatment in *P. falciparum*, we integrated the time-resolved transcriptomic and metabolomic data from the treated parasites with a genome-scale metabolic model of *P. falciparum*^11,12^. In parallel, we also coupled transcriptomic and metabolomic data from untreated parasites with the metabolic model. The model-predicted metabolism of untreated and drug-treated parasites allowed us to gain additional insights into pyrazoleamide-induced metabolic adjustments in *P. falciparum*.

### Metabolism of pyrazoleamide-treated *P. falciparum*

**Figure 4:**
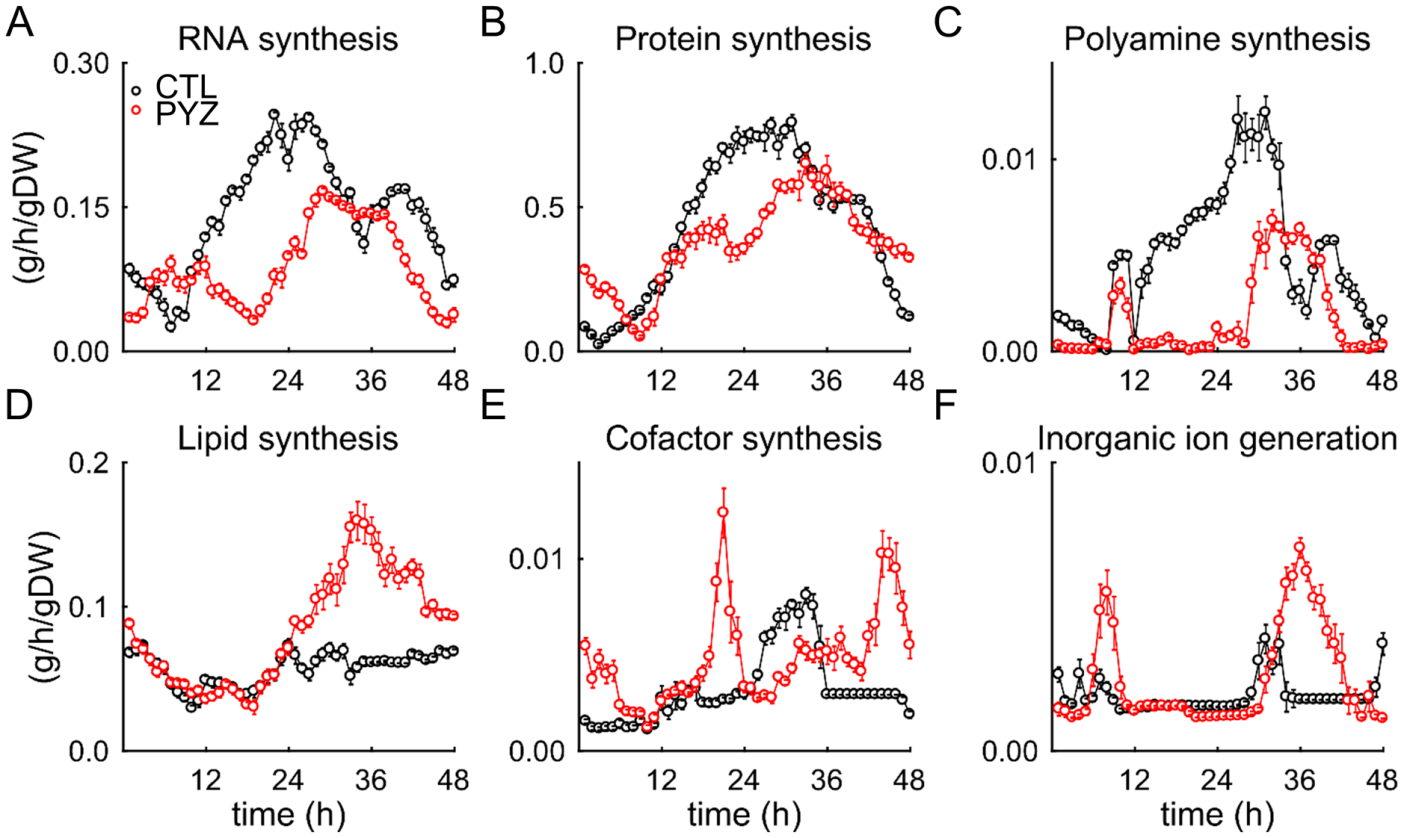
Model-predicted rates of macromolecule syntheses in untreated and pyrazoleamide-treated *P. falciparum*. (**A**) Rate of RNA synthesis. (**B**) Rate of protein synthesis. (**C**) Rate of polyamine synthesis. (**D**) Rate of lipid synthesis. (**E**) Rate of cofactor synthesis. (**F**) Rate of inorganic ion generation. The error bars denote one standard error of the mean for 10 simulations performed after adding Gaussian noise, with zero mean and 10% standard deviation in the transcriptomic data under untreated and drug-treated conditions. *CTL* parasites maintained in pure RPMI medium, *gDW* gram dry weight of the parasite, *PYZ* parasites maintained in RPMI medium with a sublethal dose of a pyrazoleamide, RPMI, Roswell Park Memorial Institute.

The integration of condition-specific and time-resolved data (transcriptomic and metabolomic) with the *P. falciparum* metabolic network model provides a functional description of the changes taking place in untreated and drug-treated parasites during the IDC. Figure 4A–F show rates of macromolecules synthesized during the IDC of untreated (black markers and curves) and drug-treated (red markers and curves) parasites. Our model predicted an inhibition of RNA, protein, and polyamine synthesis in the treated parasites (Fig. 4A–C). By contrast, our model predicted an increase in rates of lipid synthesis, cofactor synthesis, and inorganic ion generation in the treated parasites (Fig. 4D–F).

To quantify net accumulation or depletion of essential macromolecules and metabolites under both conditions, we numerically integrated their synthesis rates in the absence and presence of the drug, and then computed a ratio for each metabolite (and macromolecule) using the values obtained for untreated and drug-treated parasites (denoted by Δm). Table 3 lists Δm of select essential macromolecules and metabolites. The model predicted a net accumulation of approximately 30% and 50% in DNA and phospholipids, respectively, in the treated parasites (Table 3). In contrast to DNA and phospholipid accumulation, the model predicted a net depletion of approximately 40% and 15% in RNA and protein macromolecules, respectively, in the treated parasites (Table 3). Table S4 tabulates Δm of all essential macromolecules and metabolites.

**Table 3:** Macromolecules and metabolites accumulated in untreated and pyrazoleamide-treated parasites.

Macromolecule	Total synthesized amount (g/gDW)^†^		$\Delta \text{m } = \frac{\int {\text{v}}_{\text{PYZ}}^{\text{t}}}{\int {\text{v}}_{\text{CTL}}^{\text{t}}}$
	${\int }_{\text{t=0}}^{48}{{\text{v}}}_{\text{CTL}}^{\text{t}}$	${\int }_{\text{t=0}}^{48}{{\text{v}}}_{\text{PYZ}}^{\text{t}}$	
DNA	0.8	1.1	1.33
RNA	6.8	4.2	0.61
Protein	20.8	17.7	0.85
Phospholipid	2.7	4.0	1.49
**Metabolite**	**Total synthesized amount (mmol/gDW)**^**†**^		$\Delta {\text{m}}$
Ammonium	84.7	273.2	3.22
Protoheme	134.8	304.6	2.26
Pyridoxal 5-phosphate	167.2	294.0	1.76
Putrescine	31.1	15.1	0.49
Fe^3+^	42.7	19.5	0.46
Spermidine	72.9	15.6	0.21

*gDW* gram dry weight of the parasite.

^†^We computed the total accumulated amount of a macromolecule (or metabolite) by numerically integrating their synthesis rate (v) over the 48-h intraerythrocytic developmental cycle under untreated (CTL) or pyrazoleamide (PYZ)-treated conditions.

Vaidya et al.^10^ found that treatment of malaria parasites by lethal doses of pyrazoleamide compounds disrupts its sodium ion homeostasis, which is essential for phosphate uptake via PfPiT^29^. Our model predicted inhibition of PfATP4 and PfPiT activities in the pyrazoleamide-treated parasites (Fig. S1). As compared to untreated conditions, our integrated data and model analysis predicted substantially less efflux of cytosolic protons from the pyrazoleamide-treated parasites under steady-state conditions (Fig. S1). Interestingly, several experiments^5,7,10^ performed using lethal doses of sodium ion homeostasis disruptors have observed an increase in cytosolic pH in the treated parasites. Note that our model predictions do not suggest that cytosolic pH decreases in parasites exposed to sublethal doses of PA21A092, but rather indicate that transport of protons is minimal across the parasitic plasma membrane under steady-state conditions. Together, these results suggest an alteration in the homeostasis of sodium ions and protons in parasites treated with a sublethal dose of PA21A092.

Pyrazoleamide treatment caused temporal upregulation in expression of the gene encoding aquaglyceroporin (Table S1: Sheet 1), which transports ammonia (or water) across the parasite’s plasma membrane^16^, suggesting an accumulation of ammonia (or water) in the treated parasites. In line with these observations, our model predicted an accumulation of ammonium, the ionized form of ammonia, in the treated parasites (Δm = 3.22, Table 3). The PfPdx1 enzyme of *P. falciparum* synthesizes pyridoxal 5-phosphate (PLP) from ammonia, glyceraldehyde 3-phosphate, and ribose 5-phosphate^30^, which is also captured in our model simulations (Δm = 1.76, Table 3). Specifically, in parasites treated with a sublethal dose of PA21A092, the synthesis of PLP increased 30 h post infection, prior to which the PfPdx1 activity was minimal as compared to the untreated parasites.

### Metabolic consequences of a sublethal pyrazoleamide dose in P. falciparum

**Figure 5:**
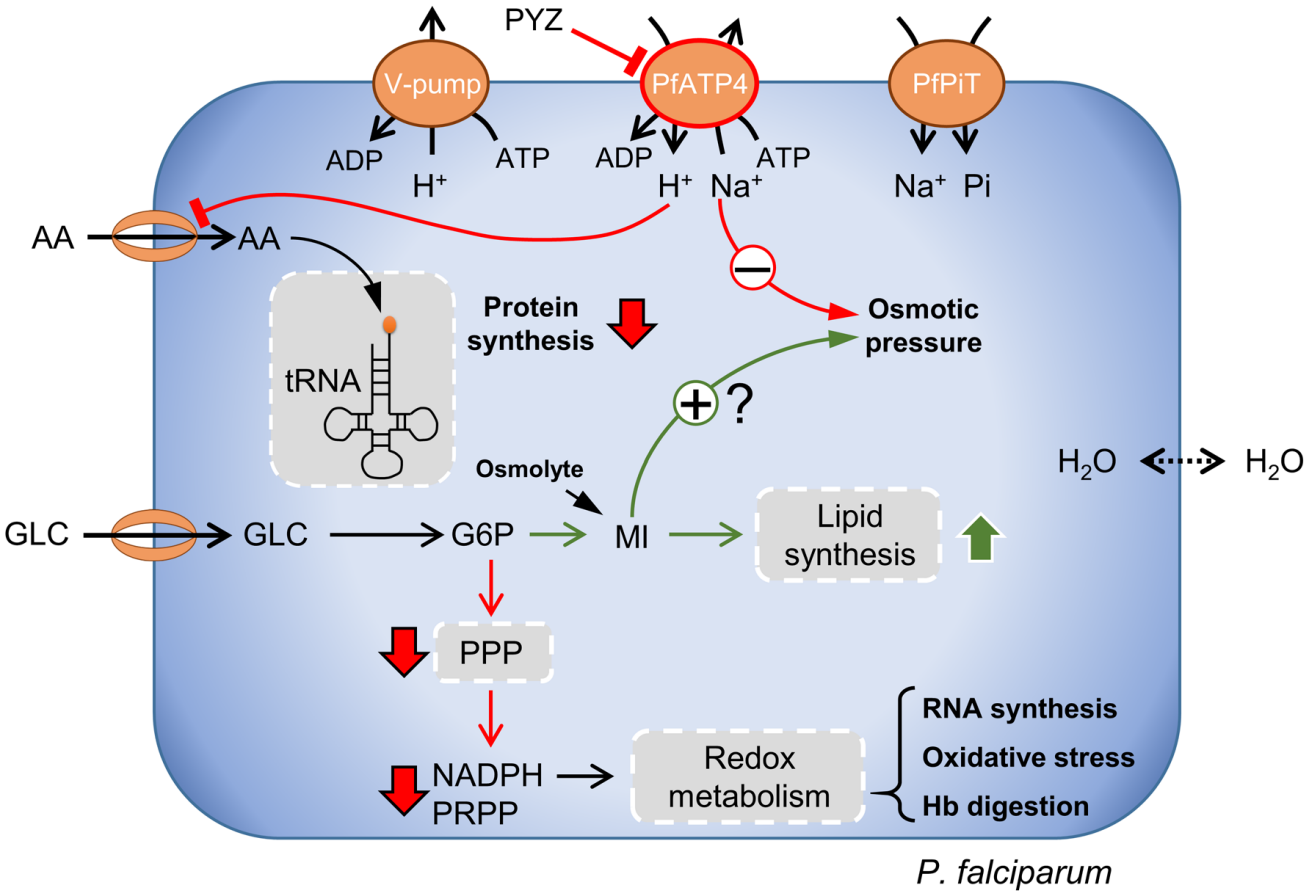
Effects of pyrazoleamide treatment in *P. falciparum*. Our integrated model analysis suggests that a sublethal dose of pyrazoleamide (PYZ) caused a reduction in PfATP4 flux, disrupting the homeostasis of sodium ions (Na^+^) and protons (H^+^) and, ultimately, reducing the osmotic pressure. The model also indicated reduced uptake of amino acids (AA), such as isoleucine, and a decreased rate of protein synthesis in drug-treated parasites. We found that the parasites adjust carbohydrate metabolism to produce an osmolyte (MI) that can counter the effects of drug treatment. However, this metabolic adjustment results in decreased flux via PPP, consequently decreasing NADPH and PRPP, and exacerbating the effects of the drug by increasing oxidative stress. Hence, our results suggest that downstream consequences of metabolic adjustments contribute to the efficacy of pyrazoleamides in *P. falciparum*. *AA* amino acid, *ADP* adenosine diphosphate, *ATP* adenosine triphosphate, *G6P* glucose 6-phosphate, *GLC* D-glucose, *Hb* hemoglobin, *MI* myoinositol, *NADPH* nicotinamide adenine dinucleotide phosphate, *Pi* inorganic phosphate, *PPP* pentose phosphate pathway, *PRPP* 5-phosphoribosyl 1-pyrophosphate, *tRNA* transfer RNA.

Figure 5 shows metabolic adjustments and adaptations in pyrazoleamide-treated *P. falciparum* as predicted by the integrated metabolic network model. We found that a sublethal dose of PA21A092 substantially inhibited PfATP4 activity during the late ring and early trophozoite stages of the IDC (Fig. S1). Because PfATP4 activity maintains the sodium ion gradient across the *P. falciparum* plasma membrane^5^, these results suggest that sublethal doses of pyrazoleamides also disrupt sodium ion homeostasis in *P. falciparum*. The model simulations predicted an inhibition of amino acid uptake in the treated parasites. Although *P. falciparum* obtains most amino acids from hemoglobin degradation, it relies on isoleucine uptake from its host to maintain growth during the IDC^31^. Accordingly, the reduction of isoleucine uptake in the treated parasites is compatible with the model-predicted 15% reduction in protein synthesis of the treated parasites (Table 3).

We found 49% more accumulation of phospholipids in pyrazoleamide-treated parasites as compared to untreated parasites (Table 3). Upon investigation of the metabolic network, we found that the treated parasites redirected flux via glucose 6-phosphate (G6P, Fig. 5) to enhance flux via myoinositol-3-phosphate synthase (MIPS, Fig. S2) and myoinositol-phosphate phosphatase (MIPP, Fig. S2). Together, these enzymes form the first and second steps of de novo synthesis of myoinositol (MI, Fig. 5). These results suggest that in drug-treated parasites the synthesis of MI—a precursor for phosphatidylinositol (PtdIns) synthesis—is increased causing an enhanced phospholipid synthesis in the treated parasites (Fig. 4D). The model simulations predict a downregulation in PPP flux as a direct consequence of this metabolic adaptation. They also showed that the downstream consequences of this metabolic adaptation, e.g., downregulated redox metabolism, increased oxidative stress, downregulated hemoglobin degradation, etc., are detrimental to the parasite and, hence, could contribute to effectiveness of pyrazoleamides in *P. falciparum*.

## Discussion

In this work, we exposed Dd2 parasites to a predetermined sublethal concentration (12 nM) of a pyrazoleamide drug (PA21A092), causing an approximately 35% reduction in growth over a single IDC. The only observable morphological change was a slight decrease in the number of developing merozoites per schizont in the drug-treated parasites at the 40-h time point (Fig. 1D). We hypothesized that when exposed to a sublethal dose, *P. falciparum* would invoke metabolic pathways to maintain growth during the IDC to counter the effects of the administered drug. To characterize *P. falciparum* responses against a sublethal dose of pyrazoleamide, we collected transcriptomic and metabolomic data during the IDC from untreated and drug-treated parasites. We classified *P. falciparum* responses into two categories: *i*) immediate or proximal response to the drug and *ii*) a downstream overall metabolic adaptation in response to the drug perturbation.

### Immediate consequences of pyrazoleamide treatment in P. falciparum

Pyrazoleamides belong to a new class of drugs that disrupt sodium ion homeostasis in *P. falciparum*^10,32^ by inhibiting the activity of the cation ATPase PfATP4. In addition to disruption of sodium ion homeostasis, pyrazoleamide treatment also causes swelling of the parasite^33^, changes in lipid composition of the parasite plasma membrane^6^, and inner membrane complexes^6^. Our analyses of the transcriptomic data revealed an approximately twofold (log_2_ scale) higher FC_0h_ of the genes encoding carbonic anhydrase and aquaglyceroporin in drug-treated parasites as compared to the untreated parasites. Carbonic anhydrase catalyzes the conversion of water and carbon dioxide into bicarbonate ions and protons, and aquaglyceroporin transports water across the parasite plasma membrane. Because the activity of carbonic anhydrase increases with pH^17^, these results suggest that expression of carbonic anhydrase is upregulated due to an increase in pH that is observed in parasites treated with a lethal dose of pyrazoleamides^10^.

Flannery et al.^7^ found that parasites treated with 10 times the IC_50_ of a pyrazoleamide drug (GNF-Pf4492) did not progress beyond the early trophozoite stages. Vaidya et al. (unpublished data) found that conditional knockdown of PfATP4 also results in arrest at the trophozoite stage. Although the pyrazoleamide dose used in our experiments is sublethal, we found that PA21A092 inhibited PfATP4 activity (Fig. S1) and caused downregulation of genes (FC_gene_ ≤ 0.5) related to parasite egress/invasion signaling in the treated parasites (Table S2).

Das et al.^6^ reported an increased accumulation of cholesterol in the parasite plasma membrane and appearance of rhoptry-like organelles in parasites treated with a lethal dose of PA21A050. Interestingly, we found substantial alterations in abundance of genes related to lipid raft and rhoptry proteins (Table S2), however, these gene-set alterations were not exclusively in the treated parasites as we also observed them in the hypoxanthine-limited parasites. In fact, we found that both hypoxanthine-limited parasites and pyrazoleamide-treated parasites shared a similar degree of gene-set alteration (Table S2). In the next Section, we discuss the mechanistic reason for this similarity between the two perturbations.

### Metabolic adaptations in pyrazoleamide-treated P. falciparum

To gain a mechanistic understanding of pyrazoleamide effects, we coupled the time-resolved data with a genome-scale model of *P. falciparum*. Our model predicted an inhibition of PfATP4 enzyme activity in the pyrazoleamide-treated parasites. We found that pyrazoleamide treatment caused approximately 15% inhibition in the rate of protein synthesis. In our model, reduced protein synthesis was primarily due to an inhibition of isoleucine uptake, which occurs via symport of protons. We found that in the treated parasites, proton exchange was minimal (Fig. S1) and was associated with the suppressed activity of membrane-bound NADP transhydrogenase (THDpp, Fig. S2), causing an inhibition in uptake of isoleucine – an amino acid essential for parasite growth^31^.

In addition to protein synthesis inhibition, we found substantially suppressed RNA synthesis activity in the treated parasites (Δm = 0.61, Table 3). Upon investigation of the metabolic network, we found that the activity of orotate phosphoribosyltransferase (ORPT)—an enzyme involved in synthesis of uridine 5′-triphosphate—was lower in the treated parasites as compared to the untreated parasites. The activity of ORPT requires orotate and 5-phosphoribosyl 1-pyrophosphate (PRPP), of which PRPP was limited in treated parasites due to the suppression of the PPP (Fig. 5 and Table 2). The PRPP metabolite is also a substrate of hypoxanthine phosphoribosyltransferase, producing inositol monophosphate from hypoxanthine^12^. Because our gene-set analyses revealed similarities in hypoxanthine-limited and pyrazoleamide-treated parasites (Table S2), we next compared macromolecular synthesis rates of hypoxanthine-limited parasites^12^ with pyrazoleamide-treated parasites, identifying reduced rates of polyamine and RNA synthesis under both perturbations. A closer examination of pyrazoleamide-treated parasite metabolism revealed that the limited availability of PRPP through the PPP also caused partial inhibition of hypoxanthine phosphoribosyltransferase activity in the treated parasites; inhibition of hypoxanthine phosphoribosyltransferase activity would have mimicked hypoxanthine limitation in the treated parasites, and caused activation of gene sets that are similar to the hypoxanthine-limited parasites (Table S2).

## Conclusions

To date, *P. falciparum* has developed some degree of resistance to all antimalarial drugs. Hence, to overcome resistance and maintain treatment efficacy, drug-combination therapies involving two (or more) drugs have become the frontline malaria treatment^34,35^. However, maintaining the long-term efficacies of these combination therapies is heavily influenced by the development and selection of mutations in the targeted parasite populations that allow the parasite to grow in the presences of drugs, a consideration that is difficult to a priori assess when developing or selecting combination therapies. In this study, our goal was to identify *P. falciparum* processes that could be relevant to the development of drug resistance. Towards this end, we cultured *P. falciparum* in the presence of a 12 nM pyrazoleamide dose, a drug whose prolonged exposure leads to resistance-conferring mutations in the PfATP4 enzyme^7,10^.

To characterize metabolic responses of *P. falciparum* during the IDC, we employed a genome-scale metabolic model integrating time-resolved metabolomic and transcriptomic data obtained in the absence and presence of the pyrazoleamide drug. We found that the treated parasites redirected flux via G6P to enhance synthesis of myoinositol and subsequently PtdIns, which regulates numerous cellular functions^36^ and plays a role in artemisinin resistance via phosphatidylinositol 3-kinase^37^. Moreover, because myoinositol has an osmoregulatory function in different species^38–40^, it can directly counter the effects of pyrazoleamide treatment, i.e., dysregulation of sodium ion homeostasis. Together, these results suggest that the mechanism underlying drug-resistance development in *P. falciparum* is specific to the administered drug. Therefore, rationally motivated drug-combination therapies that are designed to treat malaria and suppress drug-specific resistance development would be more efficacious in the long-term as compared to drug-combination therapies determined using their treatment efficacy alone.

## Materials and methods

### Synchronous culture of P. falciparum

We cultured the Dd2 strain of *P. falciparum* in human O-positive erythrocytes using methods and conditions similar to those described previously^11,12,41^. Briefly, we maintained parasite-infected erythrocytes in Roswell Park Memorial Institute (RPMI) 1640 medium (Gibco, Gaithersburg, MD) supplemented with 20 mM HEPES, 12.5 µg/mL hypoxanthine, 25 µg/mL gentamicin, 0.5 µM R-lipoic acid, 0.3% sodium bicarbonate, and 0.5% AlbuMAX II (Life Technologies, Inc., Carlsbad, CA). Addition of lipoic acid in the medium allows us to control the availability of this essential nutrient rather than relying on adventitious uptake from host erythrocytes. We obtained erythrocytes as part of an Institutional Review Board (IRB) approved phlebotomy protocol (NA_00019050), and used them within two days after collection. As per the Johns Hopkins Medicine IRB X, the use of human erythrocytes under the IRB protocol (NA_00019050) is not considered human-subject research. We obtained blood samples for untreated and drug-treated experiments on two different days. Prior to the experiments, we depleted the procured blood of white blood cells (WBCs). Towards this end, we first removed the buffy coat following two rounds of density gradient centrifugation and then used a NEO High Efficiency Leukocyte Reduction Filter (Haemonetics) to remove any remaining WBCs. Polymerase chain reaction using primers specific for the gene encoding 16S ribosomal RNA (5′-GGAGCAAACAGGATTAGATACCC and 5′-CACCATCTGTCACTCTGTTAACC) confirmed the absence of contaminating mycoplasma.

### Pyrazoleamide dose determination

To determine the optimal concentration of PA21A092 that will allow us to assess the effect of drug on parasite processes without prematurely killing the parasites, we seeded highly synchronized ring-stage parasites (2–6 h) at 5% parasitemia and 2% hematocrit (HCT) in a 96-well flat bottom plate and grew the cultures in the presence of different concentrations of PA21A092 (8–24 nM) at 37 °C for 40 h. We used 0.1% DMSO as the no-drug control and 1 µM chloroquine as a positive control. After 40 h, we diluted the treated cultures 1:10 in drug-free media and cultured for another ~ 30 h. At this point, we quantified the parasite growth using SYBR-Green I (Invitrogen) and NxT flow cytometer as described previously^19^. Based on the forward and side scatter, intact erythrocytes were gated initially. A single-cell population was selected based on forward scatter. Finally, parasitemia was obtained by gating for SYBR-Green-positive erythrocytes.

### Sample collection for metabolomic and transcriptomic profiling

We collected samples of parasites treated with the pyrazoleamide (PYZ) drug for comparison to samples collected in the absence of the drug (CTL). For each sample set, we passed 300 mL of synchronized parasite culture through a magnetically activated cell sorting (MACS) XS column (Miltenyi Biotec, Auburn, CA) to seed four flasks, serving as the source for quadruplicate measurements of transcriptomic and metabolomic data. Each flask had 75 mL of culture at 2% HCT. We monitored the parasite culture via blood smears until 0–2 h post-merozoite invasion, at which point (time 0 of the experiment) we replaced the culture medium with fresh medium containing 12 nM PA21A092 (for the PYZ sample set) or drug-free medium (for the CTL sample set). Beginning at time 0, we collected 10.5 mL of the re-suspended parasite culture from each flask every 8 h until the 48-h time point and pelleted the erythrocytes by centrifugation at 400 g for 5 min. Following aspiration of the media, we transferred 100 µL and 50 µL of the cell pellets to 1.5-mL tubes for metabolomic and transcriptomic profiling, respectively. We flash-froze the tubes in liquid nitrogen and stored them at –80 °C. For each sample set (PYZ and CTL), we sent quadruplicate samples of the cell pellets collected at six time points (0, 8, 16, 24, 32, and 40 h) to Metabolon, Inc. (Durham, NC) for metabolite analysis, and quadruplicate samples of the cell pellets collected at seven time points (0, 8, 16, 24, 32, 40, and 48 h) to the Johns Hopkins Genomic Analysis and Sequencing Core Facility for microarray analysis using an Agilent microarray chip (AMADID 037237; Santa Clara, CA). We deposited the transcriptomic data in the National Center for Biotechnology Information Gene Expression Omnibus repository (GEO accession number: GSE176469).

In parallel, we maintained four flasks with 50 mL of uRBCs at 2% HCT in media containing PA21A092 to serve as controls for the PYZ-iRBC samples, and four additional flasks with no drug to serve as controls for the CTL-iRBC samples. Following the above-mentioned procedure, we collected, centrifuged, and aspirated 7 mL of uninfected culture from each flask. We transferred 100 µL of the cell pellets to 1.5-mL tubes, and immediately flash-froze the tubes and stored them at –80 °C. We sent quadruplicate samples of these control cultures collected at six time points to Metabolon, Inc. for each of the two conditions (PYZ and CTL) for metabolomic analysis. We did not send the 48 h samples for metabolomic analysis. We show the metabolomic data as provided by Metabolon, Inc. in Table S5.

To evaluate the impact of PA21A092 treatment on parasite growth, we assessed parasitemia by a blood-smear test at every time point. At 40 h, we transferred 20 µL of the pelleted iRBCs from the four drug-treated flasks to 25-cm^2^ flasks and diluted them to 2% HCT with fresh blood and 5 mL drug-free medium. We determined parasitemia in these diluted cultures by NxT flow cytometry at 64 h and compared them to untreated controls.

### Global analyses of data

We used the built-in MATLAB function *clustergram* to perform HCA and identify substantial alterations in metabolite and gene abundances due to pyrazoleamide treatment. Within clustergram, we implemented Ward’s algorithm using a Euclidean distance metric to group metabolites and genes with similar temporal profiles between the two conditions, i.e., untreated and drug treated. For all analyses pertaining to metabolomic data, we only included metabolites having a robust signal (raw count > 1,000) across all the replicates and time points under both conditions.

To compute FC_0h_ of genes under a given condition, we used the built-in MATLAB function *bootstrp* to generate 1,000 bootstrap samples from quadruplicate samples of the dataset for each gene and time point. We then computed a fold change in expression of a gene at each time point with respect to 0 h for all the bootstrap samples, i.e., ${{F}}{{C}}_{{i}}^{{t}} \, {=} \, \left\{{{a}}_{{i}}^{{t}}/{{a}}_{{i}}^{0}{:} \, \forall{ i} \, \epsilon {{N}}\right\}$. Here, N denotes the total number of bootstrap samples, ${\text{a}}_{0}$ and ${\text{a}}_{\text{t}}$, respectively, represent abundance levels of the gene at 0 and t hours of the experiment from any of the four replicates. We then computed FC_0h_ of a gene by:

1 $${\text{F}}{\text{C}}_{\text{0h}}\text{ = }\frac{1}{{\text{N}}_{\text{tp}}}\sum_{\text{j=1}}^{{\text{N}}_{\text{tp}}}{\text{F}}{\text{C}}_{\text{j}}$$

where N_tp_ denotes the number of sampled time points during the IDC and ${\text{F}}{\text{C}}_{\text{j}}$ represents the median value of ${\text{F}}{\text{C}}_{\text{i}}^{\text{t}}$ across all bootstrap samples. To compute FC_gene_ of each gene due to a perturbation, e.g., pyrazoleamide treatment or hypoxanthine limitation, we generated 1,000 bootstrap samples, based on gene-expression data obtained from quadruplicates samples of untreated and drug-treated parasites for all time points, using the built-in MATLAB *bootstrp* function. Afterwards, we computed FC_gene_ using the following:

2 $${\text{F}}{\text{C}}_{{\text{gen}}{\text{e}}}\text{=} \frac{{\text{g}}_{\text{treatment}}}{{\text{g}}_{\text{control}}}$$

where g_control_ and g_treatment_ denote the median expression value of a gene in the untreated and drug-treated parasites, respectively. To compute FC_IDC_ of each metabolite, we followed the bootstrap procedure described in Tewari et al.^41^, and to estimate FC_0h_ and identify an internal standard (a sphingomyelin metabolite), we used the procedure described in Tewari et al.^22^. Table S6 lists the criterion (ζ value) for selecting the identified internal metabolite.

To perform gene-set analysis, we obtained a mapping of *Plasmodium* genes to the 509 gene sets available in the MPMP database^18^. For each gene set under each perturbation, we counted genes with |FC_IDC_|≥ twofold and denoted the result as a fraction of the total number of genes (N_genes_). For the results shown in Table 1, we only considered gene sets with more than 10 genes.

### Predicting effects of pyrazoleamide treatment on P. falciparum metabolism

To capture PfATP4-associated effects, we modified the *P. falciparum* network model^11^ to include the metabolic reaction for PfATP4 as described by Spillman et al.^32^. We used the built-in COBRA toolbox^42^ function *addReaction* to add the metabolic reaction of PfATP4 and assigned the *Plasmodium* gene PF3D7_1211900 to the reaction. The activity of PfATP4 is essential to maintain a sodium ion gradient, which in turn is required for phosphate uptake via PfPiT in *P. falciparum*^29^. We also included the metabolic reaction of PfPiT, as described in Saliba et al.^29^, to capture the effects of the sodium ion gradient on phosphate uptake in *P. falciparum*. We did not include the sodium-proton exchanger in the model because the gene (PF3D7_1303500) encoding this exchanger is not essential in *P. falciparum*^43^. The PfATP4-associated drugs cause pH changes in the treated parasites, presumably by the activity of V-type proton ATPases^32^. However, *P. falciparum* can extrude protons via potassium-dependent (PF3D7_1456800) and -independent transporters (PF3D7_1235200) as well. In our model, we encompassed all these reactions by including a proton transporter to extrude protons from the parasite (Fig. S1).

To simulate the effects of pyrazoleamide on *P. falciparum* metabolism, we used previously described methods^11,12,44^. Briefly, we integrated the time-resolved transcriptomic and metabolomic data obtained from untreated and pyrazoleamide-treated Dd2 parasites with the *P. falciparum* metabolic network model. To capture the effects of the drug, we assumed that pyrazoleamide treatment caused a 50% reduction in the biomass of the treated parasites as compared to untreated parasites. We used the built-in MATLAB function *trapz* to numerically integrate the simulated rates of essential macromolecules and metabolites (Table 3).

## Supplementary Information

Supplementary Figures.

Supplementary Table S1.

Supplementary Table S2.

Supplementary Table S3.

Supplementary Table S4.

Supplementary Table S5.

Supplementary Table S6.

## Data Availability

The experimental datasets generated and analyzed during the current study are included with this manuscript. The MATLAB^©^ scripts used to generate the computational results can be obtained from corresponding authors on reasonable request.
