# Supplementary material for: Metabolic adjustments of blood-stage Plasmodium falciparum in response to sublethal pyrazoleamide exposure
Source: Sci Rep. 2022 Jan 21;12:1167. doi: 10.1038/s41598-022-04985-7 (PMC8782945; doi:10.1038/s41598-022-04985-7)
Supplement: Supplementary file 1 — Supplementary Figures. [file 41598_2022_4985_MOESM1_ESM.pdf]

Electronic Supplementary Material for the paper:

Metabolic Adjustments of Blood-Stage *Plasmodium falciparum* in Response to Sublethal Pyrazoleamide Exposure

By

Shivendra G. Tewari[#], Bobby Kwan, Rubayet Elahi, Krithika Rajaram, Jaques Reifman, Sean T. Prigge, Akhil B. Vaidya, Anders Wallqvist[#]

[#]Corresponding author. Email(s): stewari@bhsai.org or sven.a.wallqvist.civ@mail.mil

This PDF file includes: Figures S1 and S2.

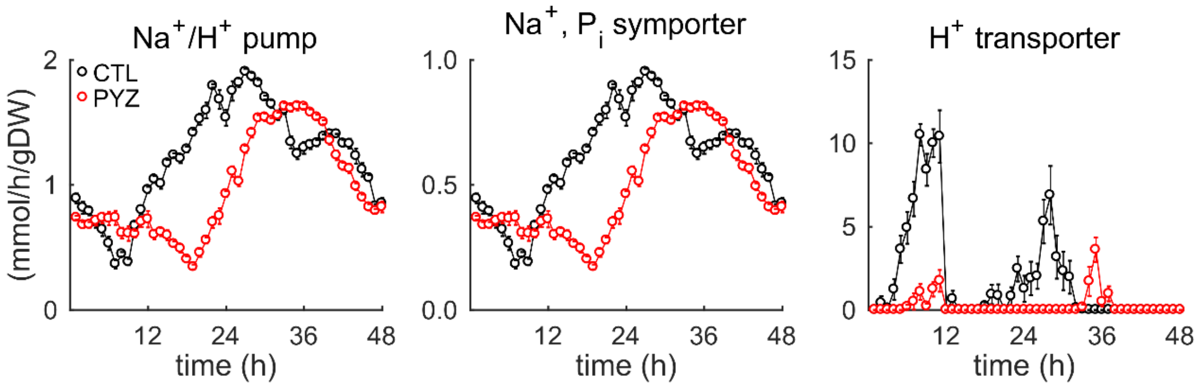

Figure S1. Model-predicted fluxes of Na^+/H^+ pump (PfATP4), Na^+, Pi symporter (PfPiT), and proton (H^+) transporter in untreated (black markers and curve) and PA21A092-treated (red markers and curve) *Plasmodium falciparum*. The error bars denote one standard error of the mean for 10 simulations performed after adding Gaussian noise, with zero mean and 10% standard deviation in the transcriptomic data under untreated and drug-treated conditions. Abbreviations: CTL, parasites maintained in pure RPMI medium; gDW, gram dry weight of the parasite; PYZ, parasites maintained in RPMI medium with a sublethal dose of PA21A092, a pyrazoleamide drug; RPMI, Roswell Park Memorial Institute.

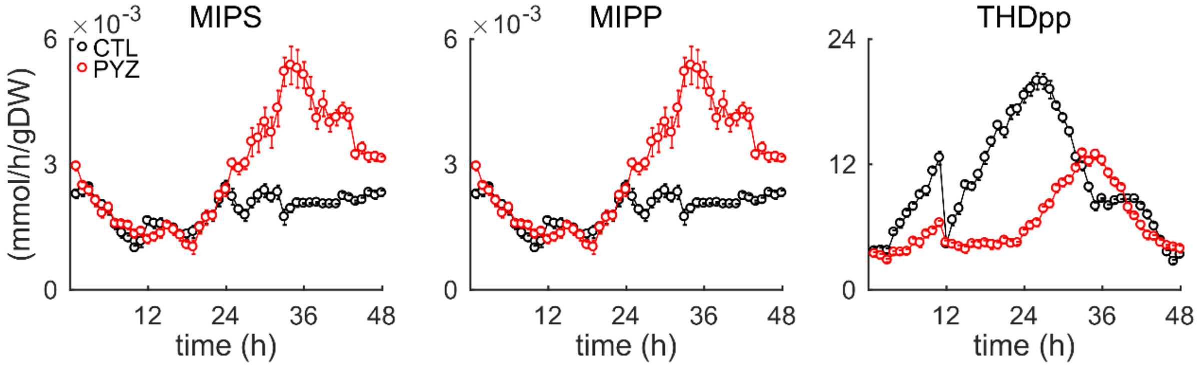

Figure S2. Model-predicted fluxes of myoinositol-3-phosphate synthase (MIPS), myoinositol-phosphate phosphatase (MIPP), and nicotinamide adenine dinucleotide phosphate transhydrogenase (THDpp) in untreated (black markers and curve) and PA21A092-treated (red markers and curve) *Plasmodium falciparum*. The error bars denote one standard error of the mean for 10 simulations performed after adding Gaussian noise, with zero mean and 10% standard deviation in the transcriptomic data under untreated and drug-treated conditions. Abbreviations: CTL, parasites maintained in pure RPMI medium; gDW, gram dry weight of the parasite; PYZ, parasites maintained in RPMI medium with a sublethal dose of PA21A092, a pyrazoleamide drug; RPMI, Roswell Park Memorial Institute.
